# QuickStats

**Published:** 2015-05-15

**Authors:** FROM THE NATIONAL CENTER FOR HEALTH STATISTICS

**Figure:**
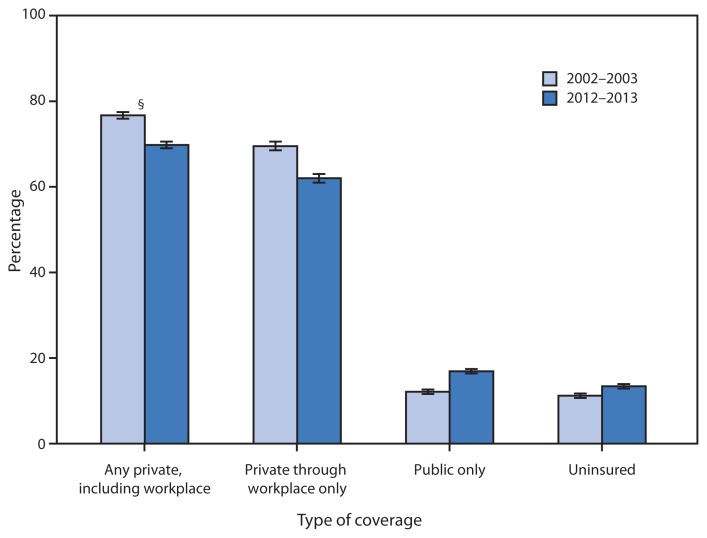
Health Insurance Coverage Among Adults Aged 55–64 Years, by Type of Coverage* — National Health Interview Survey, United States, 2002–2003 and 2012–2013^†^ * Information on health insurance coverage is collected at the time of interview. Three of the four categories (any private, including workplace; public only [Medicare, Medicaid, military, and state/local government plans]; and uninsured) are mutually exclusive, but might not sum to 100% because of rounding. ^†^ Estimates are based on household interviews of a sample of the civilian noninstitutionalized U.S. population and are derived from the National Health Interview Survey family core. ^§^ 95% confidence interval.

In 2012–2013, persons aged 55–64 years were less likely to have private health insurance coverage (69.8%) than persons in the same age group in 2002–2003 (76.7%); persons in the 2012–2013 age group also were less likely to have private coverage through the workplace (62.0%) than persons in the same age group in 2002–2003 (69.5%). Also, in 2012–2013, a greater percentage aged 55–64 years had only public health insurance coverage (16.9%) than in 2002–2003 (12.1%) and a greater percentage were uninsured (13.4%) than in 2002–2003 (11.2%).

**Source:** Health, United States, 2014 with special feature on the health of the current 55–64 year age group who within the next 10 years will enter the Medicare program. Available at http://www.cdc.gov/nchs/hus.htm.

**Reported by:** Virginia M. Freid, MS, VFreid@cdc.gov, 301-458-4220; Mary Ann Bush, MS.

